# A phase 1 trial of the MEK inhibitor selumetinib in combination with pembrolizumab for advanced or metastatic solid tumors

**DOI:** 10.1007/s10637-024-01428-0

**Published:** 2024-03-14

**Authors:** Maxime Chénard-Poirier, Aaron R. Hansen, Martin E. Gutierrez, Drew Rasco, Yan Xing, Lin-Chi Chen, Heng Zhou, Andrea L. Webber, Tomoko Freshwater, Manish R. Sharma

**Affiliations:** 1 Centre intégré de cancérologie du CHU de Québec - Université Laval, 2250 Blvd Henri-Bourassa, Quebec, QC G1J 5B3 Canada; 2 Princess Margaret Cancer Centre, Toronto, ON Canada; 3 John Theurer Cancer Center at Hackensack University Medical Center, Hackensack, NJ USA; 4 South Texas Accelerated Research Therapeutics, LLC (START), San Antonio, TX USA; 5 City of Hope National Medical Center, Duarte, CA USA; 6 Merck & Co., Inc., Rahway, NJ USA; 7 START Midwest, Grand Rapids, MI USA

**Keywords:** Clinical trial, MEK, Selumetinib, Pembrolizumab, Solid tumors

## Abstract

MEK inhibitors have immunomodulatory activity and potential for synergistic activity when combined with PD-1 inhibitors. We evaluated selumetinib (inhibitor of MEK1/2) plus pembrolizumab (anti‒PD-1 antibody) in patients with advanced/metastatic solid tumors. In this phase 1b study, adults with previously treated advanced/metastatic solid tumors received pembrolizumab 200 mg intravenously every 3 weeks plus selumetinib on days 1‒14 per 3-week cycle (2 weeks on/1 week off); selumetinib dosing began at 50 mg orally twice daily with escalation in 25 mg increments for ≤ 35 cycles. Primary endpoints were dose-limiting toxicities (DLTs), adverse events (AEs), and treatment discontinuations due to AEs. Thirty-two patients were enrolled. Dose escalation was completed up to selumetinib 125 mg twice daily. The target DLT rate of 30% was not reached at any dose level. In the selumetinib 100 mg group, 2/11 patients (18.2%) experienced DLTs (n = 1 grade 3 diarrhea, n = 1 grade 3 fatigue). In the selumetinib 125 mg group, 3/14 (21.4%) experienced DLTs (n = 1 grade 2 retinal detachment, n = 1 grade 3 retinopathy, n = 1 grade 3 stomatitis). Dose-related changes in pharmacokinetic exposures were observed for selumetinib and *N*-desmethyl selumetinib up to 100 mg (saturation at 125 mg). Two patients achieved partial responses (1 each with selumetinib 75 mg and 125 mg) for an objective response rate of 6%. The study was stopped early because of insufficient efficacy. Although the target DLT rate was not reached at any dose level and no new safety signals were identified, selumetinib plus pembrolizumab had limited antitumor activity in this population. Trial registration: ClinicalTrials.gov, NCT03833427.

**Supplementary Information:**

The online version contains supplementary material available at 10.1007/s10637-024-01428-0.

## Introduction

The mitogen-activated protein kinase (MAPK) pathway plays a key role in cell cycle regulation, including mechanisms of organ development and tissue homeostasis via mitogen-activated extracellular signal-regulated kinase 1/2 (MEK1/2), receptor tyrosine kinases, RAS and RAF family members, and extracellular signal-regulated kinase 1/2 [1]. Somatic mutations at multiple points within this pathway can lead to cancer [2]. MEK is an attractive therapeutic target as one of the main downstream effectors in the MAPK pathway. Selumetinib is an orally available selective inhibitor of MEK1/2 that is approved in the United States for the treatment of pediatric patients aged 2 years or older with neurofibromatosis type 1 with symptomatic inoperable plexiform neurofibromas [3]. Approval of selumetinib was based on phase 1 and 2 studies that demonstrated efficacy and that selumetinib had no excessive, cumulative, or irreversible toxic effects in this population [4, 5].

Combination therapies involving inhibitors of programmed cell death protein 1 (PD-1) or programmed cell death ligand 1 (PD-L1) are standard of care treatments across multiple advanced solid tumor types [6]. Preclinical evidence suggested that MEK inhibitors have immunomodulatory activity and the potential for improved activity when combined with PD-(L)1 inhibitors [7]. Some preliminary evidence of antitumor activity was suggested with the MEK inhibitor cobimetinib plus the PD-L1 inhibitor atezolizumab in patients with heavily pretreated metastatic colorectal cancer, non‒small-cell lung cancer, or melanoma [8]. In patients with previously untreated, *BRAF*-mutated advanced melanoma, early reports indicated that the addition of the PD-L1 inhibitor atezolizumab to cobimetinib and vemurafenib in the phase 3 IMspire150 study [9] or the PD-1 inhibitor pembrolizumab to trametinib and dabrafenib in the phase 1/2 KEYNOTE-022 study [10, 11] improved outcomes compared with MEK/BRAF kinase inhibition alone. Triplet therapy with an immune checkpoint, MEK, and BRAF kinase inhibitor significantly improved progression-free survival (PFS) compared with doublet therapy in the IMspire150 study and numerically improved PFS, duration of response, and overall survival (OS) in the KEYNOTE-022 study. Subsequent analyses from the IMspire150 and KEYNOTE-022 studies reported negative results [12, 13].

Based on the promising preclinical findings and preliminary results from the IMspire150 and KEYNOTE-022 clinical studies described above, the current phase 1b MK-5618-001 study (ClinicalTrials.gov, NCT03833427) was conducted to evaluate the safety and tolerability of selumetinib plus pembrolizumab in patients with advanced or metastatic solid tumors and to establish a recommended phase 2 dose of selumetinib when used in combination with pembrolizumab. The study also evaluated the pharmacokinetics of selumetinib and the antitumor activity of this combination.

## Methods

This study was done in accordance with local and/or national regulations and the ethical principles in the Declaration of Helsinki. An institutional review board or independent ethics committee approved the study protocol before the study was initiated at each site. Patients provided written informed consent before entering the study.

### Patients

Eligible patients were ≥ 18 years of age and had histologically or cytologically confirmed advanced or metastatic solid tumors with progression on, or intolerance to, all other treatments known to confer benefit. Other inclusion criteria included measurable disease as determined by Response Evaluation Criteria in Solid Tumors (RECIST) version 1.1, an Eastern Cooperative Oncology Group performance status of 0 or 1, and adequate organ function. Patients were ineligible if they had received chemotherapy, definitive radiation, or biological cancer therapy ≤ 4 weeks (≤ 2 weeks for palliative radiation) prior to the first dose of study treatment or if they were still experiencing grade ≥ 2 adverse events from a cancer therapy administered > 4 weeks earlier. Other exclusion criteria included clinically active central nervous system metastases, carcinomatous meningitis, active infection requiring therapy, history of noninfectious pneumonitis requiring steroids or current pneumonitis, active autoimmune disease requiring systemic treatment in the last 2 years, diagnosis of immunodeficiency or receipt of immunosuppressive therapy ≤ 7 days before treatment allocation, and grade > 1 peripheral neuropathy or paresthesia at baseline.

### Study design

This was a phase 1b, open-label, multicenter, multiple-dose, dose-escalation study. Patients received pembrolizumab 200 mg intravenously every 3 weeks in combination with oral selumetinib. The recommended phase 2 dose for selumetinib monotherapy is 75 mg orally twice daily administered on a continuous basis [14]. At this dose, enhanced MEK inhibition is proposed to be associated with greater response, as well as an escalation in dose-dependent adverse events. To mitigate dose-dependent adverse events associated with MEK inhibition, this study chose an intermittent dosing schedule for selumetinib (2 weeks on/1 week off) and began treatment at a dose below the recommended phase 2 dose. The starting dose of selumetinib was 50 mg twice daily on days 1 to 14 of each 3-week treatment cycle, which was escalated in 25 mg increments up to a prespecified maximum of 300 mg twice daily on days 1 to 14 of each cycle. Treatment was continued until documented radiographic disease progression, as assessed by the investigator per RECIST version 1.1 and then confirmed by modified RECIST for immune-based therapeutics (iRECIST) [15], unacceptable toxicity, intercurrent illness, investigator decision, or completion of 35 treatment cycles (~2 years). Patients with unconfirmed disease progression may have continued treatment at the discretion of the investigator until progression was confirmed using iRECIST; if repeat imaging did not confirm progressive disease per iRECIST, as assessed by the investigator, and the patient continued to be clinically stable, study treatment could have continued.

At least 3 patients were enrolled at each dose level; the modified toxicity probability interval (mTPI) design [16], with a target dose-limiting toxicity (DLT) rate of ~30%, was used to identify a potential maximum tolerated dose. Dose escalation and de-escalation decisions were based on the mTPI design and were dependent on the number of patients enrolled and number of DLTs observed at the particular dose level (Online Resource 1). Dose finding was considered complete after 14 patients were enrolled at any of the tested dose levels and the decision was made to stay at that dose level.

### Assessments

Patients were monitored for DLTs (see Online Resource 2 for definition) during the first 21 days of treatment. Adverse events were monitored throughout the study and for 30 days after cessation of study treatment (90 days for serious adverse events) and were graded according to the National Cancer Institute Common Terminology Criteria for Adverse Events version 4.0. A full ophthalmic examination was performed on day 1 of cycle 2 and every 8 weeks thereafter through treatment discontinuation, as selumetinib monotherapy is known to cause ocular toxicity, including blurred vision, photophobia, cataracts, and ocular hypertension [3, 17, 18].

Plasma samples for analyzing the pharmacokinetics of selumetinib were collected on day 1 of cycle 1 (predose; 1, 2, 4, and 6 h postdose; and 8–12 h postdose [before evening dosing]). Additional predose samples were collected on days 1 and 14 of cycle 2 and on days 1 and 14 of cycle 5.

Tumor imaging was performed at screening, every 9 weeks for 12 months, every 12 weeks during months 12 to 24, and at treatment discontinuation. For patients who discontinued study treatment without disease progression, an effort was made to continue tumor imaging (same schedule as during treatment) until the start of new anticancer treatment, disease progression, pregnancy, death, withdrawal of consent, or the end of the study.

Biomarkers were assessed at baseline and included PD-L1 combined positive score (CPS), T-cell‒inflamed gene expression profile (Tcell_inf_ GEP), tumor mutational burden (TMB), microsatellite instability (MSI), and *KRAS*/*BRAF* mutations [19–21].

### Endpoints

Primary endpoints were the occurrence of DLTs, adverse events, and study treatment discontinuations due to adverse events. Pharmacokinetic parameters of selumetinib were assessed as secondary endpoints. An exploratory endpoint was the objective response rate (ORR) per RECIST version 1.1 as assessed by the investigator, defined as the proportion of patients with a confirmed complete or partial response. Biomarkers were an additional exploratory endpoint.

### Statistical analysis

The sample size was planned at 50 to 84 patients and depended on the observed DLT rate. Analyses of adverse events were based on the safety population, which included all patients who received ≥ 1 dose of study treatment. The DLT population included all patients in the safety population who completed cycle 1 without a DLT or who experienced a DLT in cycle 1. Estimates of DLT rates across dose levels in each treatment arm were analyzed using isotonic regression with the pooled adjacent violators algorithm [16]. Pharmacokinetic analyses were based on all patients who complied with the protocol sufficiently to ensure that their data would likely reflect treatment effects. Efficacy analyses were based on all patients with a baseline scan who had measurable disease by investigator assessment and who received ≥ 1 dose of study treatment. No formal hypothesis testing was planned.

## Results

### Patients

The study was conducted between March 18, 2019, and June 28, 2022. The study was terminated early because of insufficient efficacy; dose escalation of selumetinib was completed up to 125 mg. A total of 32 patients were enrolled (n = 4 in 50 mg group, n = 3 in 75 mg group, n = 11 in 100 mg group, n = 14 in 125 mg group) at 6 study sites in the United States and Canada. Patient disposition is summarized in Online Resource 3. At the database cutoff date of July 21, 2022, the median (range) duration of follow-up (defined as time from first dose to database cutoff date) was 31.2 (22.6–39.6) months overall, 39.2 (38.7‒39.6) months in the selumetinib 50 mg group, 37.3 (36.8‒37.6) months in the selumetinib 75 mg group, 33.0 (30.8‒35.7) months in the selumetinib 100 mg group, and 26.3 (22.6‒30.0) months in the selumetinib 125 mg group. Across these dose levels, patients received a median (range) of 2 (1‒7) cycles, 2 (2‒12) cycles, 4 (1‒11) cycles, and 3 (1‒35) cycles of selumetinib, respectively. Pembrolizumab was administered for a median (range) of 2 (1‒7) doses, 9 (2‒12) doses, 3 (1‒11) doses, and 3.5 (1‒35) doses, respectively.

Patient demographics and baseline disease characteristics were generally similar across the dose levels (Table 1). Overall, 22 patients (69%) were female, and median age was 56 years. Twenty-one patients (66%) had an Eastern Cooperative Oncology Group performance status of 1, and 18 patients (56%) had received ≥ 3 previous lines of therapy. The most common cancer types were breast cancer, colorectal cancer, and ovarian cancer (n = 4 each).

**Table 1:** Patient demographics and baseline disease characteristics

	**Selumetinib 50 mg + Pembrolizumab 200 mg** **n = 4**	**Selumetinib 75 mg + Pembrolizumab 200 mg** **n = 3**	**Selumetinib 100 mg + Pembrolizumab 200 mg** **n = 11**	**Selumetinib 125 mg + Pembrolizumab 200 mg** **n = 14**	**All patients** **N = 32**
Age, median (range), y	57.5 (52‒61)	52.0 (52‒61)	58.0 (25‒78)	55.0 (36‒73)	56.0 (25‒78)
< 65 y	4 (100)	3 (100)	10 (91)	12 (86)	29 (91)
≥ 65 y	0	0	1 (9)	2 (14)	3 (9)
Sex					
Male	2 (50)	1 (33)	4 (36)	3 (21)	10 (31)
Female	2 (50)	2 (67)	7 (64)	11 (79)	22 (69)
ECOG performance status					
0	1 (25)	1 (33)	4 (36)	5 (36)	11 (34)
1	3 (75)	2 (67)	7 (64)	9 (64)	21 (66)
No. of prior lines of therapy					
1	1 (25)	0	2 (18)	2 (14)	5 (16)
2	1 (25)	0	3 (27)	3 (21)	7 (22)
3	0	2 (67)	2 (18)	0	4 (13)
4	1 (25)	0	2 (18)	4 (29)	7 (22)
≥ 5	1 (25)	1 (33)	1 (9)	4 (29)	7 (22)
Missing	0	0	1 (9)	1 (7)	2 (6)
Primary diagnosis					
Breast cancer	1 (25)	1 (33)	1 (9)	1 (7)	4 (13)
Colorectal cancer	0	1 (33)	1 (9)	2 (14)	4 (13)
Ovarian cancer	0	1 (33)	1 (9)	2 (14)	4 (13)
Esophageal cancer	0	0	1 (9)	1 (7)	2 (6)
Other solid tumor	3 (75)^a^	0	7 (64)^b^	8 (57)^c^	18 (56)

All values are n (%) unless specified otherwise. Database cutoff date: July 21, 2022

*ECOG* Eastern Cooperative Oncology Group

^a^Included lung adenocarcinoma (n = 1) and carcinoma not otherwise specified (n = 2)

^b^Included duodenal adenocarcinoma, gastroesophageal junction adenocarcinoma, malignant peripheral nerve sheath tumor, prostate cancer, thymic carcinoma, thyroid cancer, and uterine epithelioid leiomyosarcoma (n = 1 each)

^c^Included anaplastic thyroid carcinoma, cervical cancer, cholangiocarcinoma, extraskeletal myxoid chondrosarcoma, gastric cancer, pancreatic adenocarcinoma, pancreatic cancer (not islets), and carcinoma not otherwise specified (n = 1 each)

### Safety

The target DLT rate of 30% was not reached at any dose level. DLTs were reported for 0 patients in the selumetinib 50 mg and 75 mg groups. In the selumetinib 100 mg group, 2 of 11 patients (18.2% [80% CI, 7.0%‒34.9%]) experienced DLTs (n = 1 grade 3 diarrhea, n = 1 grade 3 fatigue). In the selumetinib 125 mg group, 3 of 14 patients (21.4% [80% CI, 10.2%‒36.7%]) experienced DLTs (n = 1 grade 2 retinal detachment, n = 1 grade 3 retinopathy, n = 1 grade 3 stomatitis).

Treatment-related adverse events are shown by dose level and grade in Table 2. Overall, 30 of 32 patients (94%) in the safety population experienced treatment-related adverse events. The most common events were dermatitis acneiform (47%), fatigue (34%), diarrhea (31%), and maculopapular rash (22%). Twelve patients (38%) experienced grade 3 or 4 treatment-related adverse events; those occurring in > 1 patient included increased alanine aminotransferase (n = 1 in 100 mg group, n = 3 in 125 mg group), increased aspartate aminotransferase (n = 2 in 125 mg group), diarrhea (n = 1 in 50 mg group, n = 1 in 100 mg group), and retinopathy (n = 1 in 100 mg group, n = 1 in 125 mg group). Eight patients (25%) discontinued study treatment (selumetinib, pembrolizumab, or both) due to treatment-related adverse events; the only event that led to discontinuation of study treatment in > 1 patient was pneumonitis (n = 2 in 125 mg group [both selumetinib and pembrolizumab were discontinued]). Four patients had selumetinib dose reductions due to treatment-related AEs; there were no pembrolizumab dose reductions due to AEs. No treatment-related adverse events led to death.

**Table 2:** Treatment-related adverse events

**Treatment-Related Adverse Event, n (%)**	**Selumetinib 50 mg + Pembrolizumab 200 mg** **n = 4**		**Selumetinib 75 mg + Pembrolizumab 200 mg** **n = 3**		**Selumetinib 100 mg + Pembrolizumab 200 mg** **n = 11**		**Selumetinib 125 mg + Pembrolizumab 200 mg** **n = 14**		**All patients** **N = 32**	
Any grade	2 (50)		3 (100)		11 (100)		14 (100)		30 (94)	
Grade 3/4^a^	1 (25)		0		6 (55)		5 (36)		12 (38)	
Led to discontinuation of any study drug	0		1 (33)		4 (36)		3 (21)		8 (25)	
Selumetinib	0		1 (33)		4 (36)		3 (21)		8 (25)	
Pembrolizumab	0		0		2 (18)		3 (21)		5 (16)	
Led to death	0		0		0		0		0	

	**Any Grade**	**Grade 3/4**	**Any Grade**	**Grade 3/4**	**Any Grade**	**Grade 3/4**	**Any Grade**	**Grade 3/4**	**Any Grade**	**Grade 3/4**
Most common (≥ 2 patients total)										
Dermatitis acneiform	1 (25)	0	1 (33)	0	7 (64)	0	6 (43)	0	15 (47)	0
Fatigue	0	0	2 (67)	0	5 (46)	1 (9)	4 (29)	0	11 (34)	1 (3)
Diarrhea	1 (25)	1 (25)	3 (100)	0	4 (36)	1 (9)	2 (14)	0	10 (31)	2 (6)
Maculopapular rash	0	0	1 (33)	0	3 (27)	0	3 (21)	0	7 (22)	0
Dry skin	0	0	1 (33)	0	1 (9)	0	4 (29)	0	6 (19)	0
Nausea	0	0	0	0	2 (18)	0	4 (29)	0	6 (19)	0
Increased alanine aminotransferase	0	0	0	0	1 (9)	1 (9)	4 (29)	3 (21)	5 (16)	4 (13)
Dyspepsia	0	0	1 (33)	0	0	0	4 (29)	0	5 (16)	0
Rash	0	0	1 (33)	0	0	0	4 (29)	0	5 (16)	0
Vomiting	0	0	0	0	1 (9)	0	4 (29)	0	5 (16)	0
Increased aspartate aminotransferase	0	0	0	0	1 (9)	0	3 (21)	2 (14)	4 (13)	2 (6)
Decreased appetite	0	0	1 (33)	0	1 (9)	0	2 (14)	0	4 (13)	0
Pneumonitis	0	0	0	0	1 (9)	0	3 (21)	1 (7)	4 (13)	1 (3)
Hyperthyroidism	1 (25)	0	1 (33)	0	0	0	1 (7)	0	3 (9)	0
Pruritus	1 (25)	0	0	0	0	0	2 (14)	0	3 (9)	0
Stomatitis	0	0	0	0	1 (9)	0	2 (14)	1 (7)	3 (9)	1 (3)
Anemia	0	0	0	0	1 (9)	0	1 (7)	1 (7)	2 (6)	1 (3)
Increased blood creatine phosphokinase	1 (25)	0	0	0	1 (9)	1 (9)	0	0	2 (6)	1 (3)
Constipation	0	0	0	0	1 (9)	0	1 (7)	0	2 (6)	0
Hypophosphatemia	0	0	0	0	0	0	2 (14)	1 (7)	2 (6)	1 (3)
Hypothyroidism	1 (25)	0	0	0	0	0	1 (7)	0	2 (6)	0
Peripheral edema	0	0	0	0	0	0	2 (14)	0	2 (6)	0
Retinal detachment	0	0	1 (33)	0	0	0	1 (7)	0	2 (6)	0
Retinopathy	0	0	0	0	1 (9)	1 (9)	1 (7)	1 (7)	2 (6)	2 (6)
Vision blurred	0	0	0	0	0	0	2 (14)	0	2 (6)	0
Vitreous floaters	1 (25)	0	1 (33)	0	0	0	0	0	2 (6)	0

Database cutoff date: July 21, 2022

^a^There were no grade 5 treatment-related adverse events

### Pharmacokinetics

**Fig. 1:**
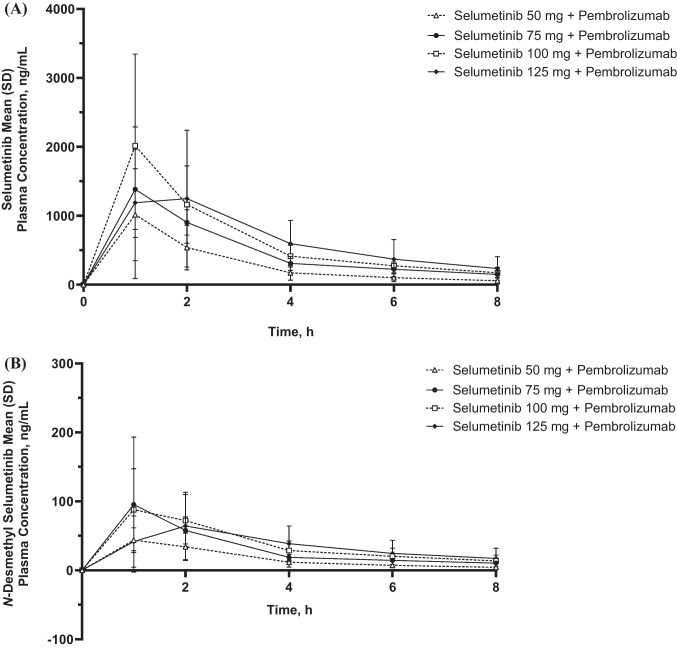
Arithmetic mean ± standard deviation plasma concentration‒time profiles for **A** selumetinib and **B** *N*-desmethyl selumetinib on day 1 of cycle 1. Database cutoff date: July 21, 2022

On day 1 of cycle 1, plasma exposures of selumetinib and its metabolite *N*-desmethyl selumetinib (area under the concentration‒time curve and maximum concentration) increased as dose increased up to 100 mg, with saturation observed at 125 mg (Table 3). On day 1 of cycle 1, plasma concentrations of selumetinib and *N*-desmethyl selumetinib peaked between 1 and 2 h postdose at each dose level and steadily declined thereafter through 8 h postdose (Fig. 1A, B). The observed t_1/2_ ranged from 2.82 h to 3.82 h for selumetinib and 3.07 h to 3.94 h for *N*-desmethyl selumetinib (Table 3).

**Table 3:** Plasma pharmacokinetic parameters of selumetinib and metabolites on day 1 of cycle 1

	**Selumetinib 50 mg + Pembrolizumab 200 mg**	**Selumetinib 75 mg + Pembrolizumab 200 mg**	**Selumetinib 100 mg + Pembrolizumab 200 mg**	**Selumetinib 125 mg + Pembrolizumab 200 mg**
Selumetinib				
n	4	3	11	14
AUC_0‒last_, h$\cdot$ng/mL	2080 (71.4)	3800 (5.7)	4660 (51.2)	4430 (57.6)
AUC_0‒12_, h$\cdot$ng/mL	2160 (73.1)	4220 (8.5)	5060 (47.7)	4760 (62.0)
C_max_, ng/mL	852 (79.5)	1400 (33.4)	1690 (90.4)	1410 (70.5)
t_last_, h	9.63 (8.00‒11.50)	8.00 (8.00‒8.00)	8.00 (7.93‒8.33)	8.03 (7.83‒11.85)
t_1/2_, h	3.35 (29.6)	3.82 (17.2)	3.12 (69.9) (n = 10)	2.82 (56.9) (n = 11)
*N*-Desmethyl selumetinib				
n	4	3	11	14
AUC_0‒last_, h$\cdot$ng/mL	128 (43.5)	229 (49.6)	260 (71.8)	235 (67.4)
AUC_0‒12_, h$\cdot$ng/mL	134 (45.5)	257 (44.0)	291 (62.8)	257 (71.8)
C_max_, ng/mL	46.9 (23.4)	76.7 (105.4)	78.6 (99.3)	67.7 (64.7)
t_last_, h	9.63 (8.00‒11.50)	8.00 (8.00‒8.00)	8.00 (7.93‒8.33)	8.03 (7.83‒11.85)
t_1/2_, h	3.52 (54.8)	3.07 (37.0)	3.94 (140.7) (n = 10)	3.16 (49.5) (n = 11)

Data are geometric mean (% geometric coefficient of variation) except for t_last_ (median [range]). Database cutoff date: July 21, 2022

*AUC*_*0‒last*_ Area under the concentration‒time curve from time 0 through last measurable concentration, *AUC*_*0‒12*_ Area under the concentration‒time curve from time 0 through 12 h postdose, *C*_*max*_ Maximum concentration, *t*_*last*_ Time of last measurable concentration, *t*_*1/2*_ Half-life

### Efficacy

Best overall response is shown in Table 4. No complete responses were observed. Partial responses were observed in 1 of 3 patients (33%) in the selumetinib 75 mg group and in 1 of 14 patients (7%) in the selumetinib 125 mg group; no partial responses were seen in the selumetinib 50 mg and 100 mg groups. The durations of response for these 2 patients were 6.0 months (colorectal cancer) and 25.1 months (anaplastic thyroid carcinoma), respectively. The patient with a duration of response of 25.1 months discontinued selumetinib after the first cycle and completed 35 cycles of pembrolizumab. Progressive disease was observed in 2 of 4 patients (50%) in the selumetinib 50 mg group, 1 of 3 patients (33%) in the selumetinib 75 mg group, 4 of 11 patients (36%) in the selumetinib 100 mg group, and 6 of 14 patients (43%) in the selumetinib 125 mg group. A reduction of ≥ 30% in target lesion size was observed in 1 patient receiving selumetinib 75 mg (colorectal cancer), 1 receiving selumetinib 100 mg (esophageal cancer), and 3 receiving selumetinib 125 mg (anaplastic thyroid carcinoma, pancreatic adenocarcinoma, and cervical cancer) (Online Resource 4).

**Table 4:** Objective response rates per response evaluation criteria in solid tumors version 1.1 by investigator

	**Selumetinib 50 mg + Pembrolizumab 200 mg** **n = 4**	**Selumetinib 75 mg + Pembrolizumab 200 mg** **n = 3**	**Selumetinib 100 mg + Pembrolizumab 200 mg** **n = 11**	**Selumetinib 125 mg + Pembrolizumab 200 mg** **n = 14**	**All patients** **N = 32**
Objective response rate	0	1 (33)	0	1 (7)	2 (6)
Best overall response					
Complete response	0	0	0	0	0
Partial response	0	1 (33)	0	1 (7)	2 (6)
Stable disease	1 (25)	1 (33)	5 (45)	7 (50)	14 (44)
Progressive disease	2 (50)	1 (33)	4 (36)	6 (43)	13 (41)
No assessment^a^	1 (25)	0	2 (18)	0	3 (9)

All values are n (%). Database cutoff date: July 21, 2022

^a^Includes patients without postbaseline assessment at the database cutoff date

### Biomarkers

The prevalence of biomarkers at baseline is shown in Online Resource 5. Thirteen patients (41%) had tumors with PD-L1 CPS ≥ 10, 16 patients (50%) had Tcell_inf_ GEP low tumors, 24 patients (75%) had non–TMB-high tumors (< 10 mut/Mb), 25 patients (78%) had microsatellite stable (MSS) tumors, and no patient had known microsatellite instability-high/mismatch repair deficient tumors. Five patients (16%) had *KRAS* mutations; none had *BRAF* mutations. Biomarkers in the patient with a PR in the selumetinib 75 mg group included PD-L1 CPS 90, Tcell_inf_ GEP non-low, TMB-high (≥ 10 mut/Mb), MSS, and *KRAS*/*BRAF* wild type (Online Resource 6). The patient with a PR in the selumetinib 125 mg group did not have any biomarker data available.

## Discussion

This phase 1b study investigated the safety and tolerability of the combination of selumetinib and pembrolizumab in patients with advanced or metastatic solid tumors after intolerance to or disease progression during all available approved therapies. Dose escalation of selumetinib was completed up to 125 mg, at which point the study was terminated by the sponsor. The target DLT rate of 30% was not reached at any dose level, suggesting that the maximum tolerable dose level for selumetinib exceeds 125 mg. Pharmacokinetic results showed that plasma levels of selumetinib and *N*-desmethyl selumetinib increased in a dose-related manner up to 100 mg, and that saturation was met at 125 mg. No new safety concerns were observed, and the safety profile of the combination of selumetinib plus pembrolizumab was consistent with the reported safety profiles of the individual agents. Selumetinib plus pembrolizumab demonstrated limited antitumor activity, with partial responses observed in only 6% of patients. One of the 2 patients with a partial response had a tumor with TMB ≥ 10 mut/Mb, which is a known predictive biomarker for response to pembrolizumab. Biomarker data were available for 1 of the 2 PRs and were consistent with what would be expected for response to pembrolizumab monotherapy. Efficacy findings were not sufficient to pursue further development of this combination.

Early reports of efficacy across multiple trials indicated that the combination of an anti-PD-(L)1 antibody with one or more inhibitors along the MAPK-MEK-BRAF signaling axis may be a promising therapeutic approach in select advanced solid tumors [8–11]. Following initiation of the MK-5618-001 study, clinical trials that evaluated MEK/BRAF kinase inhibitors combined with PD-(L)1 inhibitors for *BRAF*-mutant advanced melanoma published more mature, negative results. The second interim analysis of the IMspire150 study showed no significant improvement in OS with triplet therapy (atezolizumab, cobimetinib, and vemurafenib) compared with MEK/BRAF kinase inhibition alone [12] and, in the phase 3 COMBI-i study, the addition of the PD-1 inhibitor spartalizumab to trametinib plus dabrafenib did not significantly improve PFS and led to increased toxicity compared with trametinib plus dabrafenib [22]. In a phase 1 study, DLTs of dermatitis (n = 5), QTc prolongation (n = 1), and arthralgias (n = 1) were reported among 5 patients treated with the triplet combination of pembrolizumab, cobimetinib, and vemurafenib, and the safety findings led to early closure of the study [23].

Similar findings have been reported for *BRAF*-wild type or *BRAF*-unselected melanoma and other advanced solid tumors. In the phase 3 IMspire170 study, patients with *BRAF* wild-type advanced melanoma had no PFS benefit with cobimetinib plus atezolizumab relative to pembrolizumab alone (HR, 1.15 [95% CI, 0.88‒1.50]), and rates of adverse events and treatment discontinuations or dose modifications due to adverse events were higher with combination therapy than monotherapy [24]. In the KEYNOTE-022 study, limited antitumor activity (ORR, 17%) was demonstrated with trametinib plus pembrolizumab in patients with *BRAF* wild-type advanced melanoma or advanced solid tumors regardless of *BRAF* mutation; safety was manageable, although there was no control arm [13]. Lastly, the phase 3 IMblaze370 study evaluated cobimetinib plus atezolizumab versus monotherapy with atezolizumab or the multikinase inhibitor regorafenib in patients with predominantly microsatellite-stable, unresectable locally advanced or metastatic colorectal cancer [25]. Overall survival was similar among the 3 treatment groups and rates of serious adverse events and treatment discontinuations due to adverse events were more common with combination therapy than monotherapy [25].

Interpretation of the current study results is limited by the small sample size, particularly given that cohort expansion was not initiated. Since the pharmacokinetic data showed saturation at the 125 mg dose, it was concluded that further escalation of the selumetinib dose was unlikely to improve efficacy. Due to early study termination and small sample size, no further biomarker analysis is planned.

In conclusion, selumetinib plus pembrolizumab had manageable safety but did not demonstrate sufficient antitumor activity in patients with advanced or metastatic solid tumors. Accumulating evidence suggests that despite a mechanistic rationale for combining MAPK inhibition with immune checkpoint inhibition, such regimens have modest efficacy and limited utility for patients with advanced or metastatic solid tumors.

### Supplementary information

Below is the link to the electronic supplementary material.

Supplementary file1 (PDF 474 mb)

## Data Availability

Merck Sharp & Dohme LLC, a subsidiary of Merck & Co., Inc., Rahway, NJ, USA (MSD) is committed to providing qualified scientific researchers access to anonymized data and clinical study reports from the company’s clinical trials for the purpose of conducting legitimate scientific research. MSD is also obligated to protect the rights and privacy of trial participants and, as such, has a procedure in place for evaluating and fulfilling requests for sharing company clinical trial data with qualified external scientific researchers. The MSD data sharing website (available at: http://engagezone.msd.com/ds_documentation.php) outlines the process and requirements for submitting a data request. Applications will be promptly assessed for completeness and policy compliance. Feasible requests will be reviewed by a committee of MSD subject matter experts to assess the scientific validity of the request and the qualifications of the requestors. In line with data privacy legislation, submitters of approved requests must enter into a standard data-sharing agreement with MSD before data access is granted. Data will be made available for request after product approval in the US and EU or after product development is discontinued. There are circumstances that may prevent MSD from sharing requested data, including country or region-specific regulations. If the request is declined, it will be communicated to the investigator. Access to genetic or exploratory biomarker data requires a detailed, hypothesis-driven statistical analysis plan that is collaboratively developed by the requestor and MSD subject matter experts; after approval of the statistical analysis plan and execution of a data-sharing agreement, MSD will either perform the proposed analyses and share the results with the requestor or will construct biomarker covariates and add them to a file with clinical data that is uploaded to an analysis portal so that the requestor can perform the proposed analyses.
