# Supplementary material for: A phase 1 trial of the MEK inhibitor selumetinib in combination with pembrolizumab for advanced or metastatic solid tumors
Source: Invest New Drugs. 2024 Mar 14;42(3):241–51. doi: 10.1007/s10637-024-01428-0 (PMC11164811; doi:10.1007/s10637-024-01428-0)
Supplement: Supplementary file 1 — Supplementary file1 (PDF 474 mb) [file 10637_2024_1428_MOESM1_ESM.pdf]

SUPPLEMENTAL MATERIAL

A Phase 1 Trial of the MEK Inhibitor Selumetinib in Combination With Pembrolizumab for Advanced or Metastatic Solid Tumors

Maxime Chénard-Poirier,[1] Aaron R. Hansen,[2] Martin E. Gutierrez,[3] Drew Rasco,[4] Yan Xing,[5] Lin-Chi Chen,[6] Heng Zhou,[6] Andrea L. Webber,[6] Tomoko Freshwater,[6] Manish R. Sharma[7]

[1]Centre intégré de cancérologie du CHU de Québec - Université Laval, Quebec, QC, Canada; [2]Princess Margaret Cancer Centre, Toronto, ON, Canada; [3]John Theurer Cancer Center at Hackensack University Medical Center, Hackensack, NJ, USA; [4]South Texas Accelerated Research Therapeutics, LLC (START), San Antonio, TX, USA; [5]City of Hope National Medical Center, Duarte, CA, USA; [6]Merck & Co., Inc., Rahway, NJ, USA; [7]START Midwest, Grand Rapids, MI, USA

Corresponding author: Maxime Chénard-Poirier
Centre intégré de cancérologie du CHU de Québec - Université Laval
2250 Blvd Henri-Bourassa
Quebec, QC, G1J 5B3
Canada
Email: maxime.chenard-poirier.1@ulaval.ca

Online Resource 1. Dose-Finding Rules per Modified Toxicity Probability Interval Design

	No. of Patients Evaluable for DLTs at Current Dose											
**No. of Patients With ≥1 DLT**	3	4	5	6	7	8	9	10	11	12	13	14
0	E	E	E	E	E	E	E	E	E	E	E	E
1	S	S	S	E	E	E	E	E	E	E	E	E
2	D	S	S	S	S	S	S	S	E	E	E	E
3	DU	DU	D	S	S	S	S	S	S	S	S	S
4		DU	DU	DU	D	D	S	S	S	S	S	S
5			DU	DU	DU	DU	DU	D	S	S	S	S
6				DU	DU	DU	DU	DU	DU	D	S	S
7					DU	DU	DU	DU	DU	DU	DU	D
8						DU	DU	DU	DU	DU	DU	DU
9							DU	DU	DU	DU	DU	DU
10								DU	DU	DU	DU	DU
11									DU	DU	DU	DU
12										DU	DU	DU

	No. of Patients Evaluable for DLTs at Current Dose											
**No. of Patients With ≥1 DLT**	3	4	5	6	7	8	9	10	11	12	13	14
13											DU	DU
14												DU

D, de-escalate to the next lower dose; DLT, dose-limiting toxicity; DU, current dose is unacceptably toxic; E, escalate to the next higher dose; S, stay at current dose.

Online Resource 2. Definition of Dose-Limiting Toxicity[a]

Grade 4 nonhematologic toxicity (not laboratory)

Grade 4 hematologic toxicity lasting ≥7 d except

- Grade 4 thrombocytopenia of any duration
- Grade 3 thrombocytopenia associated with clinically significant bleeding

Any nonhematologic adverse event of grade 3 severity (not laboratory) except

- Grade 3 fatigue lasting ≤3 d
- Grade 3 diarrhea, nausea, or vomiting without use of antiemetics or antidiarrheals per standard of care
- Grade 3 rash without use of corticosteroids or anti-inflammatory agents per standard of care

Any grade 3 or 4 nonhematologic laboratory value if

- Clinically significant medical intervention is required to treat the patient, or
- The abnormality leads to hospitalization, or
- The abnormality persists for >1 wk, or
- The abnormality results in a drug-induced liver injury

Grade 3 or 4 febrile neutropenia

- Grade 3: absolute neutrophil count <1000/mm^3 with a single temperature of >38.3°C (101°F) or sustained temperature of ≥38°C (100.4°F) for >1 h
- Grade 4: absolute neutrophil count <1000/mm^3 with a single temperature of >38.3°C (101°F) or sustained temperature of ≥38°C (100.4°F) for >1 h, with life-threatening consequences and urgent intervention indicated

Any prolonged delay (>2 wk) in initiating cycle 2 study treatment due to a treatment-related toxicity

Any treatment-related toxicity that causes the patient to discontinue treatment during cycle 1

Missing >25% of selumetinib doses as a result of drug-related adverse event(s) during the first treatment cycle

Grade 5 toxicity

Cardiac disorders including

- Absolute decrease in left ventricular ejection fraction >10% compared with baseline and below the institution’s lower limit of normal
- Grade ≥3 left ventricular systolic dysfunction
- Other cardiac disorders grade ≥3

Vascular disorders including

- Grade ≥3 hypertension requiring >1 drug or more intensive therapy
- Grade 4 hypertension

Eye disorders including

- Grade ≥3 retinopathy or retinal detachment, confirmed by ophthalmic examination
- Retinal vein disorder, including retinal vein occlusion, confirmed by ophthalmic examination
- Visual disturbances without ocular (retinal) changes
- Grade ≥3 blurred vision, flashing lights, or floaters
- Other (not listed above)
  - Grade ≥3 for >21 consecutive days
  - Grade 4 confirmed by ophthalmic examination

[a]All toxicities were graded using National Cancer Institute Common Terminology Criteria for Adverse Events version 4.0 based on investigator assessment. The dose-limiting toxicity window of observation was during the first 21 days of study treatment. The occurrence of any of the toxicities listed in this table was considered a dose-limiting toxicity if assessed by the investigator to be possibly, probably, or definitely related

to study treatment, excluding toxicities clearly not related to study treatment (eg, disease progression, environmental factors, or unrelated trauma).

Online Resource 3. Patient disposition. DLT, dose-limiting toxicity; PD, progressive disease. Database cutoff date: July 21, 2022.

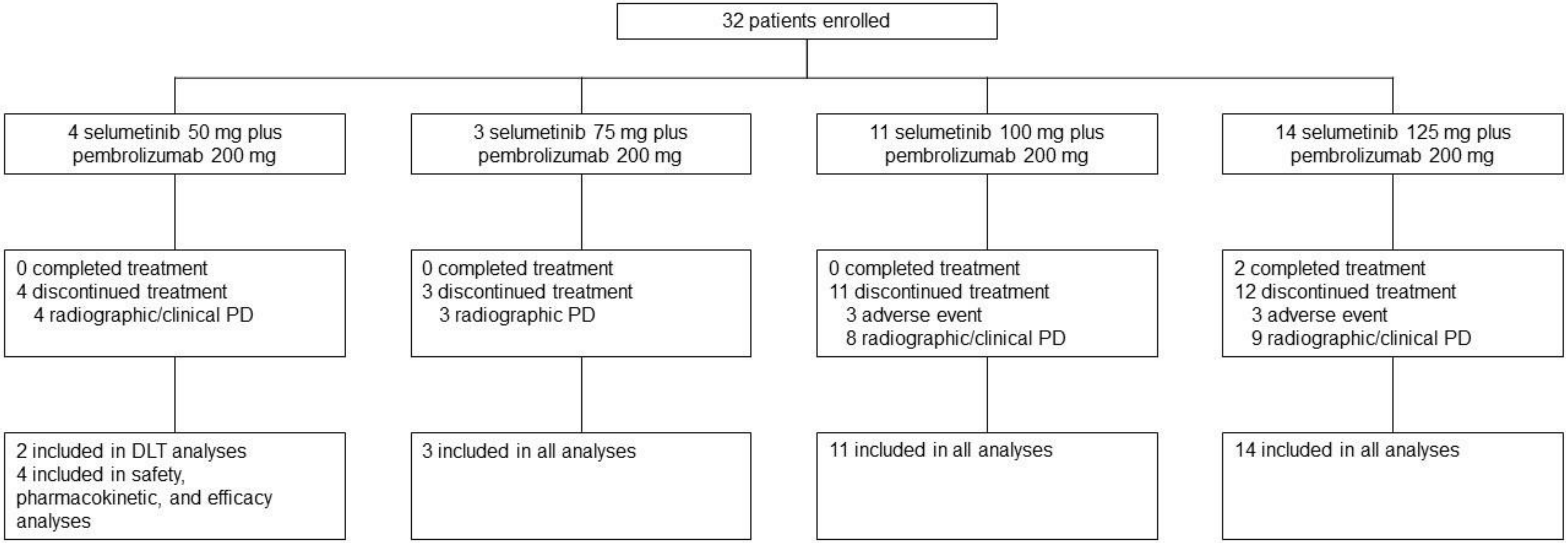

Online Resource 4. (**A**) Change from baseline over time and (**B**) best percentage change from baseline in target lesions per RECIST version 1.1 by investigator for all patients with ≥1 postbaseline target lesion. (**C**) Time on study treatment and response per RECIST version 1.1 by investigator assessment for all patients. The last assessment before discontinuation of study drug (or within 1 cycle of discontinuation of study drug) is plotted as 'Patient off study drug' in the spider plots. *Represents patients with a confirmed partial response. RECIST, Response Evaluation Criteria in Solid Tumors. Database cutoff date: July 21, 2022.

A.

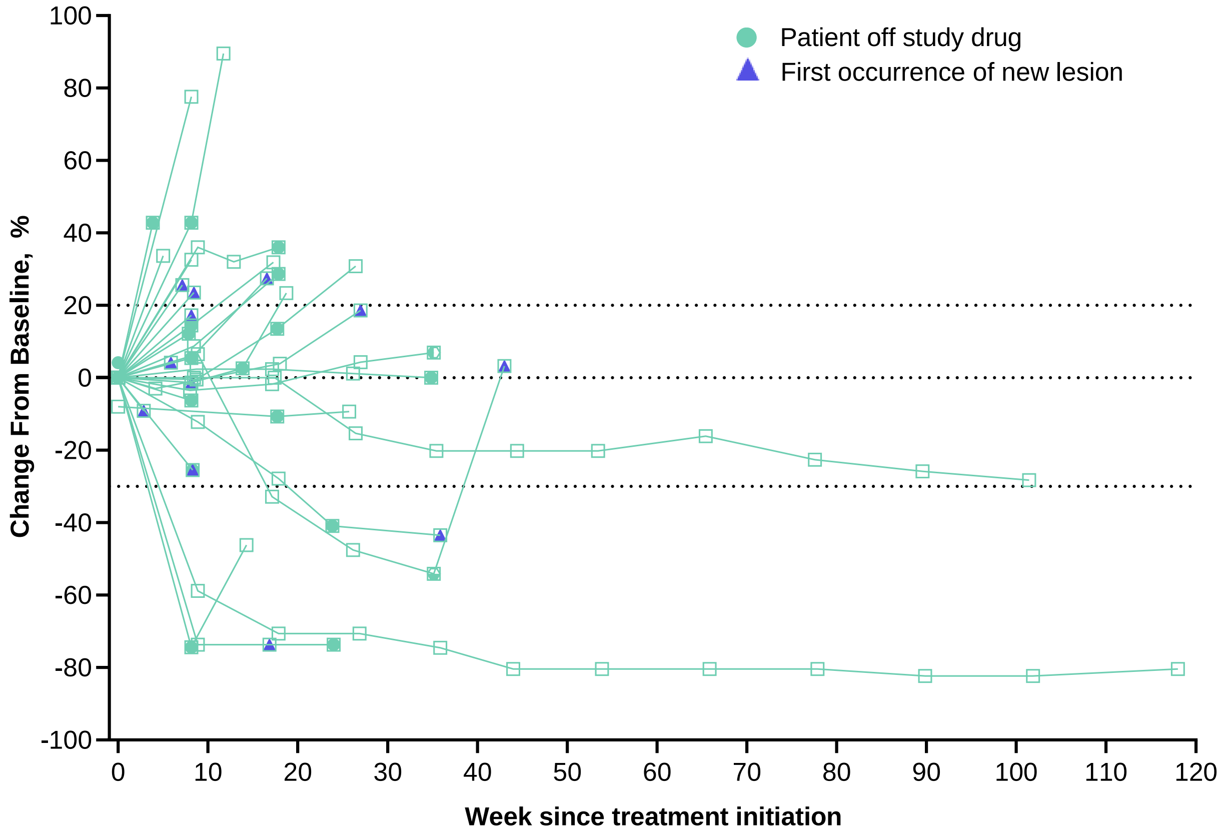

B.

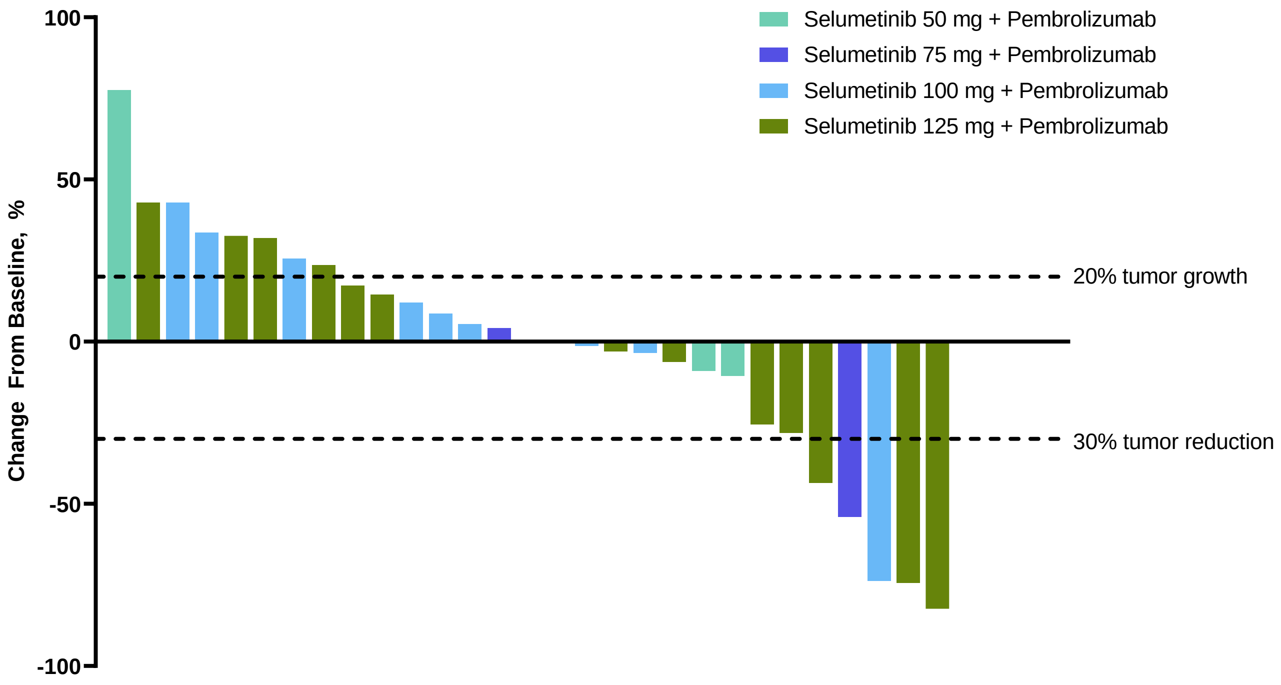

Selumetinib 50 mg + Pembrolizumab
Selumetinib 75 mg + Pembrolizumab
Selumetinib 100 mg + Pembrolizumab
Selumetinib 125 mg + Pembrolizumab
100
50
0
-50
-100
Change From Baseline, %
20% tumor growth
30% tumor reduction

C.

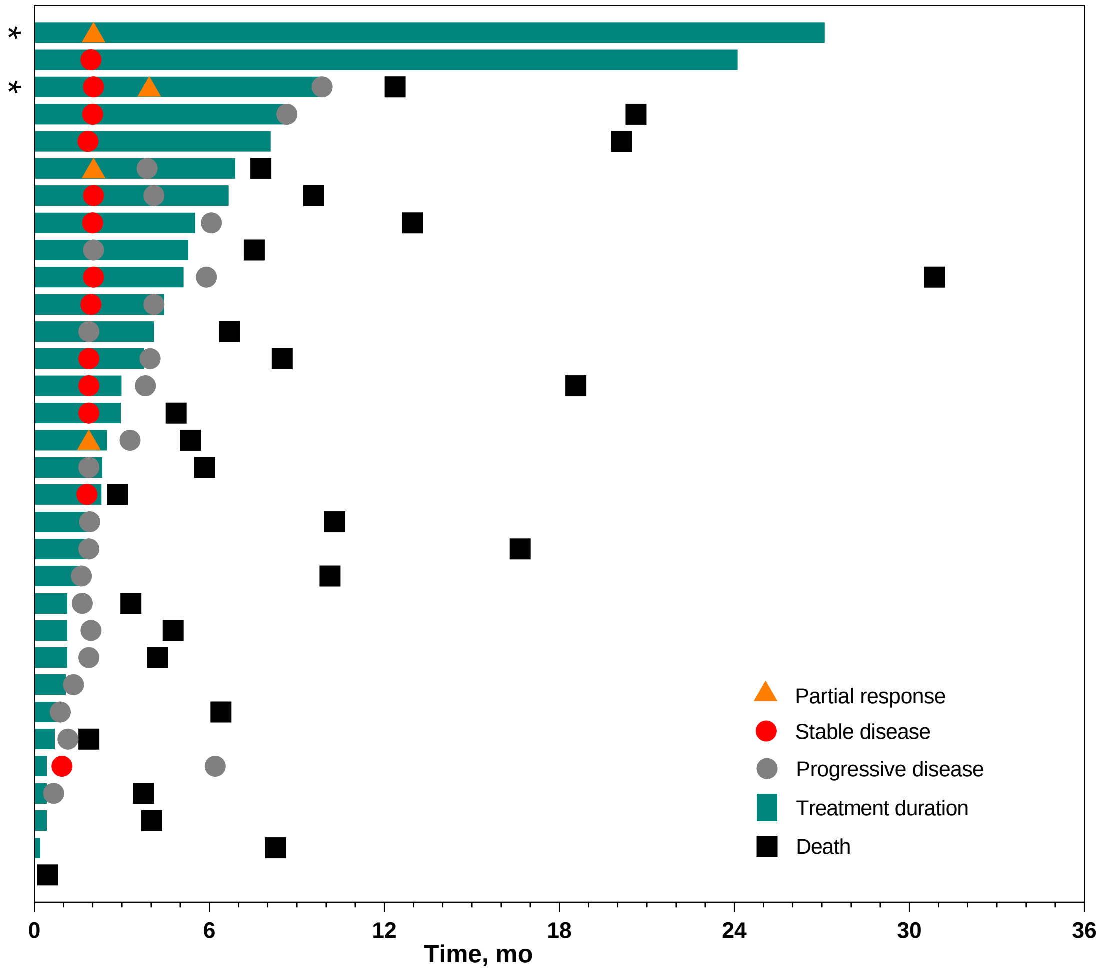

*
*
Partial response
Stable disease
Progressive disease
Treatment duration
Death
0
6
12
18
24
30
36
Time, mo

Online Resource 5. Biomarker Prevalence at Baseline

	All Patients<br>N = 32
PD-L1 CPS[a]	
<1	6 (19)
1–9	8 (25)
≥10	13 (41)
Other (NSCLC)	1 (3)
Not Evaluable	2 (6)
Missing	2 (6)
$Tcell_{inf}$ GEP[b]	
Non-low	12 (38)
Low	16 (50)
Missing	4 (13)
TMB[c]	
TMB-H (≥10 mut/Mb)	1 (3)
Non–TMB-H (<10 mut/Mb)	24 (75)
Missing	7 (22)
MSI	
MSI-H	0
MSS	25 (78)
Missing	7 (22)
KRAS	
Mutation	5 (16)
Wild Type	20 (63)
Missing	7 (22)
BRAF	
Mutation	0
Wild Type	25 (78)
Missing	7 (22)

Data are n (%).

CPS, combined positive score; $Tcell_{inf}$ GEP, T-cell–inflamed gene expression profile; MSI, microsatellite instability; MSI-H, microsatellite instability-high; MSS, microsatellite stable; NSCLC, non–small-cell lung

cancer; PD-L1, programmed cell death ligand 1; TMB, tumor mutational burden; TMB-H, tumor mutational burden-high.

[a]One patient with NSCLC had a PD-L1 tumor proportion score of 1.

[b]A GEP cutoff of −0.318 was used for low vs non-low.

[c]Based on TruSight Oncology 500 assay.

Online Resource 6. Relationship between biomarkers and response. (**A**) PD-L1 CPS, (**B**) $Tcell_{inf}$GEP, and (**C**) TMB. Biomarkers in the patient with a partial response in the selumetinib 75 mg group included PD-L1 CPS 90, $Tcell_{inf}$ GEP non-low, TMB-high (≥10 mut/Mb), microsatellite stable, and *KRAS/BRAF* wild type. The patient with a partial response in the selumetinib 125 mg group did not have any biomarker data available. CPS, combined positive score; PD-L1, programmed cell death ligand 1; $Tcell_{inf}$GEP, T-cell–inflamed gene expression profile TMB, tumor mutational burden. Database cutoff date: July 21, 2022.

A.

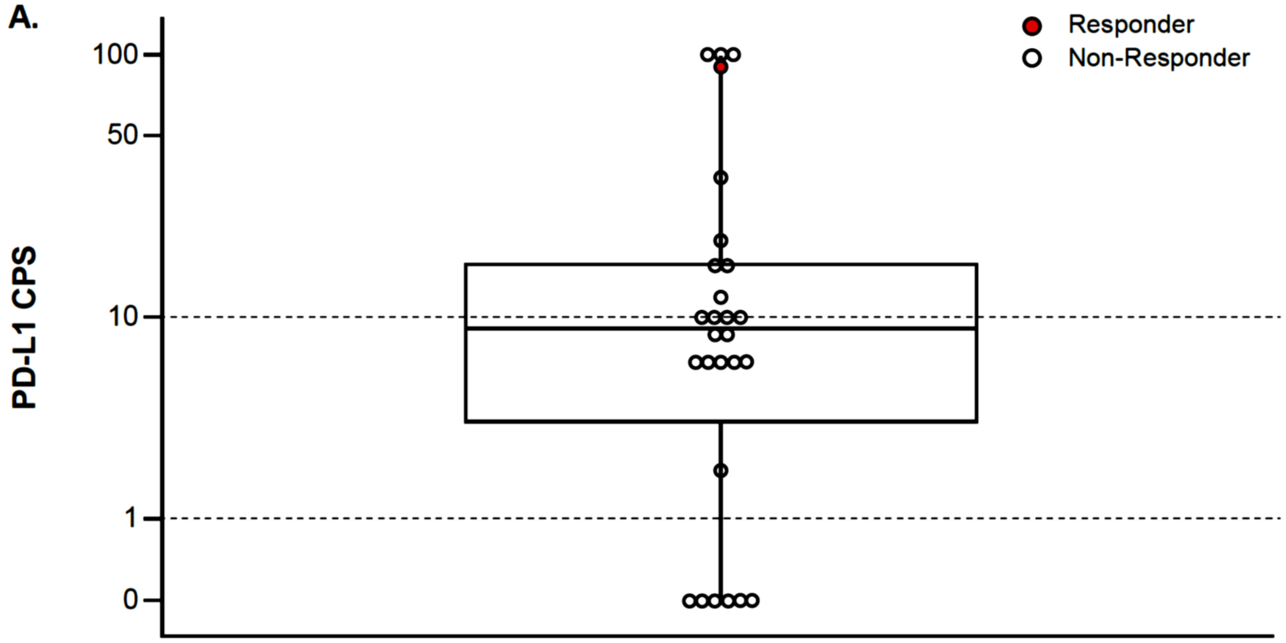

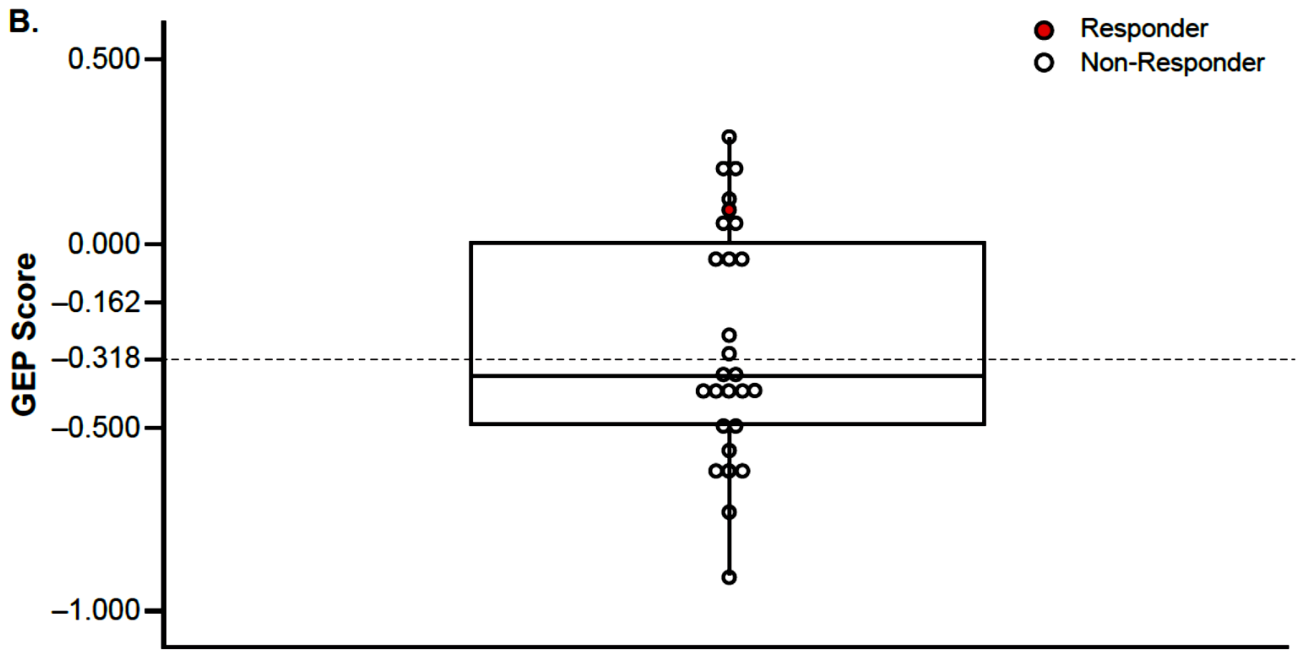
B.
0.500
0.000
–0.162
–0.318
–0.500
–1.000
GEP Score
Responder
Non-Responder

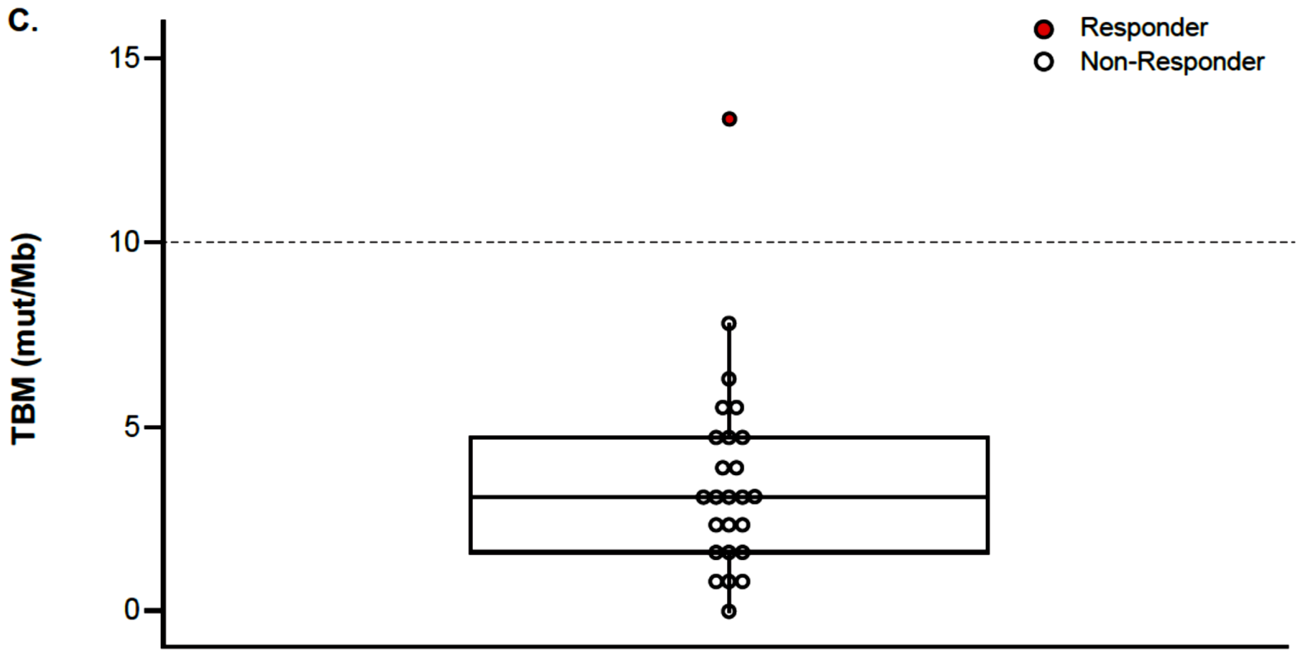
C.
15
10
5
0
TBM (mut/Mb)
Responder
Non-Responder
